# Chimeric antigen receptor for adoptive immunotherapy of cancer: latest research and future prospects

**DOI:** 10.1186/1476-4598-13-219

**Published:** 2014-09-21

**Authors:** Huan Shi, Meili Sun, Lin Liu, Zhehai Wang

**Affiliations:** Department of Oncology, Shandong Cancer Hospital and Institute, No. 440 Jiyan Road, Jinan, Shandong 250117 P.R. China; Department of Oncology, Jinan Central Hospital affiliated to Shandong University, No. 105, Jie Fang Road, Jinan, Shandong 250013 P.R. China

**Keywords:** Adoptive transfer, Chimeric antigen receptor, Gene transfer, T cells

## Abstract

Chimeric antigen receptors (CARs) are recombinant receptors that combine the specificity of an antigen-specific antibody with the T-cell’s activating functions. Initial clinical trials of genetically engineered CAR T cells have significantly raised the profile of T cell therapy, and great efforts have been made to improve this approach. In this review, we provide a structural overview of the development of CAR technology and highlight areas that require further refinement. We also discuss critical issues related to CAR therapy, including the optimization of CAR T cells, the route of administration, CAR toxicity and the blocking of inhibitory molecules.

## Introduction

Adoptive cellular therapy (ACT) has received much attention as a realistic technique for cancer treatment [1–3]. Tumor-reactive T cells can be isolated from tumor-infiltrating lymphocytes (TILs) and then expanded *in vitro* before re-infusion back into cancer patients [4]. The adoptive transfer of TILs yields a durable regression of melanoma tumors [5, 6]. However, the process by which tumor-reactive TILs are isolated and expanded is technically difficult, labor-intensive and time-consuming. Moreover, another limitation in the more widespread application of TIL therapy is the difficulty in identifying antigen-specific T cells in other cancer types.

To overcome these obstacles and to broaden the applications of ACT, gene-therapeutic approaches for the redirection of T-cells to defined tumor-associated antigens (TAAs) have been developed [7]. One sophisticated strategy involves the engineering of autologous T-cells with a chimeric antigen receptor (CAR) [8], which is composed of a specific antigen-binding moiety that is derived from the variable regions of a monoclonal antibody (mAb) and linked through a hinge and a transmembrane (TM) motif to a cytoplasmic lymphocyte-signaling moiety [9, 10]. The CARs endow T cells antigen-specific recognition, activation and proliferation in an MHC-independent manner. Current clinical trials using engineered CAR T cell therapy demonstrate clinical responses in both hematological malignancies and solid tumors [2, 11]. Here, we will provide an overview of the recent development of the CAR technology and discuss the challenges and future prospects for this pioneering approach.

### CAR binding domain

**Figure 1:**
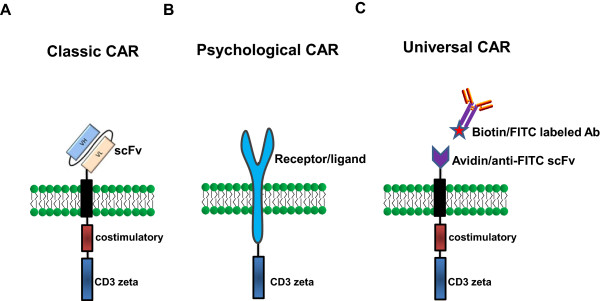
**Schematic of different chimeric antigen receptors (CARs) used to re-direct the T cell immune response. (A)** Schematic structure of second-generation classic CAR. Second-generation CARs contain one costimulatory endodomains (illustrated with CD28 or 4-1BB or OX-40 or CD27), cloned in frame with the scFv and the CD3z endodomain. **(B)** Schematic structure of physiological CAR which contains full length CD27 or NKG2D receptor fused to CD3z endodomain. **(C)** Schematic structure of universal CAR, which utilize biotin or anti-FITC scFv as binding domain fused costimulatory and CD3z endodomains.

The classic CAR consists of an extracellular antigen-recognition domain attached to an extracellular spacer/hinge domain, a TM region that anchors the receptor to the cell surface and a signaling endodomain. A scFv derived from the variable heavy chain (VH) and variable light chain (VL) regions of an antigen-specific mAb linked by a flexible linker is commonly utilized as the extracellular TAA-binding domain in most CARs (Figure 1A). The scFv retains the same specificity and a similar affinity as the full antibody from which it was derived [12]. Moreover, the small molecular size of scFvs facilitates both the genetic manipulation and expression of the CAR. Furthermore, it determines the CAR antigen specificity and binds the target protein in an MHC-independent manner. To date, the scFvs of CARs are most often derived from mouse mAbs. Human anti-mouse antibody (HAMA) responses can occur within days and can block antigen recognition by CARs. Therefore, the use of humanized [13] or fully human scFv [14] may be preferable to mouse scFv. In addition, the affinity of scFv must be considered in the design of CARs. The affinity of the scFv selected for designing a CAR also should be considered. Hudecek et al. [15] showed that increasing the affinity of a CAR enhances its T-cell effector function and recognition of tumors. However, the development of higher affinity CARs with greater anti-tumor activity could theoretically increase the risk of on-target toxicity and mandates careful safety studies in a relevant model.

The extracellular antigen-recognition domain of CARs can also be a ligand for a receptor that is expressed on tumor cells [11]. Non scFv-based ligand-binding domains have been utilized in a CAR format (Figure 1B). For example, the CD27 receptor [16], the heregulin molecule (a ligand for Her3 and Her4 receptors) [17], interleukin (IL)-13 mutein [18], vascular endothelial growth factor (anti-VEGFR2) [19], and the NKG2D receptor [20–22], have been used successfully for engineered T-cell therapy, resulting in tumor regression in vivo. Recently, a novel chimeric NKp30 CAR targeting the B7-H6 (NKp30 ligand) expressing tumor was developed [23].

To expand the applications for T cell-based immunotherapy in cancer, Tamada et al. [24] and Urbanska et al. [25] constructed similar “universal” CARs (uCAR) that utilize anti-fluorescein isothiocyanate (FITC) scFv and avidin in either a monomeric (mcAv) or dimeric (dcAv) form as binding domains fused to T-cell signaling domains, respectively (Figure 1C). These uCAR T cells recognize various cancer types when bound to FITC-labeled or biotinylated antigen-specific mAbs or scFvs, resulting in efficient target lysis, T-cell proliferation, and cytokine production. More recently, Kudo et al. [26] constructed a novel uCAR containing the high-affinity CD16 (FCGR3A) V158 variant, CD8α hinge and transmembrane domains, along with signaling domains. CD16V-based uCAR T cells have bound humanized antibodies with higher affinity and engagement of the CD16V-uCAR provoked T cell activation, exocytosis of the lytic granules and sustained proliferation. Further, the co-administration of CD16V uCAR T cells with immunotherapeutic antibodies exerted considerable antitumor activity in vivo. Importantly, the treatment of immunocompromised mice using the novel uCAR T cells plus the labeled mAbs currently in clinical use exhibited potent antitumor activity. The need for many different immune receptor genes to cover all cancers limits the feasibility of ACT, and the use of uCARs may address this issue.

### CAR targeting

Most antigens targeted by CAR-T cells are simply ‘tumor-associated’ and not ‘tumor-specific’. The potential for “on-target, off-organ” toxicity is a serious concern in CAR T-cell therapy. Thus, the judicious selection of TAAs is the first step and is critical to the success of CAR-based ACT. CD19 is the widely and successfully utilized target of CAR-modified T cells [27–29], being universally expressed by acute lymphoblastic leukemia, the most common malignancy of children, whereas its expression on non-tumor tissues is restricted to B-cells and their progenitors, but not hematopoietic stem cells. The toxicity of targeting this antigen using anti-CD19 CAR-modified T cells is limited to B cell aplasia and the consequent effects on humoral immunity, which is considered to be a tolerable side-effect of this therapy [11]. In contrast, one colon cancer patient treated with Her2/neu CAR-T cells died 5 days after the adoptive transfer; this patient died of what appears to have been a cytokine storm and respiratory failure triggered by the recognition of the low levels of antigens on lung epithelial cells [30]. These studies suggest that ideal TAAs are required by the tumor cell for survival and should show restricted expression to the tumor cell surface and otherwise non-vital tissues.

The effect of antigen density for CAR therapy is not yet well defined. It appears that CAR T cells typically target highly expressed antigens, while low antigen-expressing tumor cells are resistant to CAR T cell therapy [31, 32]. This resistance could be a limitation in their activity against tumors expressing low antigen levels. The intensity of antigen expression on target cells, however, can be increased by the administration of epigenetic modulators [32]. On the other hand, lesser sensitivity may become an advantage when the avoidance of low-level antigen expression on normal cells is desirable.

### CAR signaling

CARs are grouped into three generations of increasing costimulatory activity (Figure 1A). The first-generation CARs contain a single signaling unit that is most commonly derived from the CD3z chain or FcRg subunits [33]. However, first-generation CARs have limited clinical activity for the treatment of lymphoma, neuroblastoma, and ovarian and renal cancer [34–37] because the activation of the CAR-modified T cells induces only transient cell division and suboptimal cytokine production, and these functions fail to produce prolonged T-cell expansion and sustained antitumor effects [38].

The therapeutic success of adoptive therapy with CAR T cells depends on the appropriate costimulation of CD3z to induce full T-cell activation [39]. These CARs contain costimulatory signaling domains derived from the T cell costimulatory molecules, such as CD28, which is the molecule most commonly selected by CARs [29, 40–42]. However, other costimulatory molecules, such as 4-1BB (CD137), OX40 (CD134), ICOS and CD27, also play important roles in regulating T-cell proliferation, survival, and antitumor functions [10, 41, 43]. Notably, Porter et al. [44] described a heavily pretreated patient with chronic lymphocytic leukemia (CLL) who had a complete remission. This remission was associated with the tumor lysis syndrome following the transfer of second-generation CD19 CAR-T cells coupled with 4-1BB and CD3z signaling domains.

A special second-generation CAR developed recently separates the T cell signaling domains into two different CARs, one of which contains the costimulatory signaling domains, such as CD28 or 4-1BB, while the other CAR, with a different specificity, contains only the CD3z signaling domain. This strategy reproduces the physiological signals’ 1 and 2 checkpoints of T cell activation. Wilkie et al. [45] have tested this principle by co-expressing Her2- and MUC1-specific CARs that signal using CD3z and CD28, respectively. They found that “dual-targeted” T cells kill Her2 + tumor cells efficiently and proliferate in a manner that requires the co-expression of MUC1 and Her2 by tumor cells in vitro. Recently, Kloss et al. [46] presented a similar strategy and co-transduced T cells to express CARs targeting the prostate tumor antigens PSMA and PSCA. They showed that co-transduced T cells destroy tumors that express both antigens but do not affect tumors that express either antigen alone. Hence, the potential for “on-target” toxicity should be reduced. These findings further pave the way for testing the safety of this strategy in clinical trials.

Furthermore, combining the signaling from multiple signaling molecules, such as CD3, CD28, and CD137 (or CD134) to form a 3rd generation CAR has also been tested [47, 48]. In a clinical setting, Till et al. have reported that CARs containing three activation motifs have potent anti-tumor efficacy [49].

### CAR hinge and transmembrane

In addition to signaling domains, previous studies [50, 51] highlight the requirement of a spacer/hinge domain inserted between the scFv binding and transmembrane(TM) domain in CD3z-signaling CARs for its stable expression on the surface of T cells. A spacer or flexible hinge region domain mediates CAR flexibility and appears to be important for ensuring the suitable positioning of the binding domain during scFv-antigen interactions [51, 52]. In addition, the TM domains have significant effects on the cell surface expression of CARs and may also influence CAR function. For example, Pulè et al. [53] showed that CARs containing the CD28 TM domain result in the highest expression, while the CAR transduction with the OX40 and CD3z TM domains have intermediate and the lowest expressions, respectively. Zhang et al. [23] also showed that CD28 TM-containing chimeric NKp30 CARs often show greater surface expression than do CARs with CD3z TM, perhaps because the CD28 TM-containing CARs tend to predominantly form homodimers independent of the TCR-CD3 complex, whereas the CD3z TM-containing CARs can form heterodimers of CAR with the endogenous CD3z chain that may be limited by the TCR-CD3 complex expression. Greater expression of CD28 TM-containing CARs was shown to correlate with better functional activity.

Currently, various TM regions have been employed in CAR, including CD3z [18], FcϵRIγ [54], CD4 [55], CD7 [56], CD8 [10, 47], CD28 [10, 47], OX40 [57] and H2-Kb [58]. However, there is evidence supporting the notion that the intracellular domains, rather than the TM domains, mediate stable cell surface expression [59]. Additional comparative studies of different cytoplasmic and TM domains are required.

### Generation of CAR modified T cells

**Figure 2:**
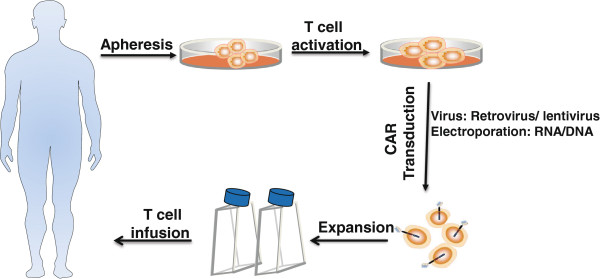
**Schema for adoptive cellular therapy with genetically modified CAR T cells.** T cells can be isolated from patient blood by apheresis, and genetically modified to express a transgene encoding a tumor-specific CAR. The genetically modified T cells are then expanded in vitro using several approaches before infusion into the patient.

Both lentiviruses and retroviruses have been widely used as gene transfer vectors, and they compose the vector system that is currently used in the majority of clinical gene therapy trials for cancer [60] (Figure 2). However, the lentiviral vectors have become more widely used and are advantageous because they mediate the efficient transduction of cells, can be used with both dividing and nondividing cells, result in long-term, stable transgene expression and appear to be less prone to gene silencing [60]. Nonviral gene transfer technologies have been explored for gene therapy. Dr. Cooper’s group [61, 62] reported a new nonviral approach for the electrotransfer of DNA plasmids using the Sleeping Beauty (SB) transposon/transposase system into primary human T cells, which resulted in efficient and stable CD19-specific CAR gene expression.

An alternative non-viral approach that does not rely on transgene integration, which uses RNA electroporation, results in transient CAR expression, precluding effective T-cell persistence beyond a week [63] (Figure 2). The use of transient CAR T cells, which require multiple injections to provide meaningful tumor responses, may reduce the destruction of normal tissues or prevent T-cell accumulations to levels that increase the risk of cytokine storms [64]. More recently, mRNA CAR T cells have mediated antitumor activity in patients with advanced solid tumors [65]. Thus, these results support the development of mRNA CAR-based strategies for cancer therapy.

### Optimization of CAR T cells

It is now clear that the adoptive transfer of the less-differentiated naive (T_N_) or central memory (T_CM_) T cell subsets is associated with superior T cell engraftment, persistence, and antitumor activity, thus correlating highly with the objective clinical responses [66]. These subsets can be enriched using cell surface molecules such as CD62L before the CAR introduction; these cells have been shown to persist to a greater extent in vivo than the more differentiated T cells [67, 68]. A recently identified stem cell-like population of T cells [69] with strong engraftment potential in peripheral blood may be more effective for ACT and is worth exploring for CAR-redirected targeting in vivo. Moreover, when common γ chain cytokines such as IL-7, IL-15, and IL-21 are added to the T cell cultures, they shift the final T cell phenotype towards that of a less-differentiated T cell type [70, 71]. Furthermore, the chemokine system plays a major role in driving T cell migration. Therefore, the expression of specific chemokine receptors that can aid in the precise trafficking of T cells to tumors have been explored, including the co-expression of CXCR2 and CCR2b on CAR-T cells [72–74].

### Route of administration of CAR T cells

Although systemic (intravenous, IV) injection is favored in clinical applications because of its ease of administration, several preclinical studies [47, 75, 76] suggest that the regional (intratumoral, IT or intraperitoneal, IP) administration of T cells may provide optimal therapeutic effects, which may be in part due to increased T-cell trafficking to the tumor.

Indeed, Dr. Maher’s group [76] showed that CAR T cells remain at the site of inoculation with minimal systemic absorption when delivered via IP or IT routes. In contrast, after IV administration, CAR T cells initially reach the lungs and then are redistributed to the spleen, liver, and lymph nodes. These findings may help to explain the development of rapid lung toxicity [30] and grades 2–4 liver toxicity [36] in Her2-specific CAR and CAIX-specific CAR T cell therapy trials. More recently, Dr. Maher’s group [77] showed that ErbB CAR T cells elicit antitumor activity in mice in the absence of detectable clinical or histologic toxicity when administered in moderate doses by the IV or IP routes. However, when large numbers of these cells are administered using the IP route, cytokine release syndrome results. In contrast, when delivered using the IT route, T-cells remain at the site of injection for several days, where they promote tumor regression but never elicit cytokine release syndrome. These findings raise the possibility that ErbB-targeted T cells may prove useful in the treatment of human malignancy provided that the dosing and route of administration are optimized carefully. To test this possibility, Dr. Maher’s group [78] recently designed a protocol for the phase I clinical testing of the intratumoral injection of CAR T cells in locally advanced or recurrent head and neck squamous cell carcinomas (Clinicaltrials.gov number: NCT01818323). Intratumoral injection may provide a safe and potentially effective management strategy for CAR therapy.

In addition, RNA CAR-electroporated T cells may be particularly suitable for regional administration, due to the transient nature of the CAR expression on the T cells [64]. Furthermore, clinical studies have shown the feasibility and safety of both the intratumoral and intraperitoneal injection of T cells [79, 80].

Overall, a local route of administration of the engineered T cells may provide the optimal therapeutic effect and decrease the potential for the “on-target, off-organ” toxicity discussed below.

### CAR toxicity

The immune-mediated recognition of targeted antigens in normal tissues is referred to as “on-target, off tumor” toxicity. “On-target” toxicity was first reported for the CAIX CAR [36], which was used to treat patients with metastatic renal cell cancer and consisted of limiting the liver enzyme elevations that were most likely caused by the CAR T cells that recognized the CAIX antigen expressed at low levels on the bile duct epithelial cells. The elimination of the normal B-cell compartment in the patients treated with CD19-specific CAR T cells represents an expected on-target toxicity that can be managed by administering intravenous immunoglobulin [27, 81].

In addition, tumor lysis syndrome (TLS) and cytokine release syndrome (CRS) were also reported in patients treated with CD19 CAR T cells [27, 44, 81]. TLS is a group of metabolic abnormalities that results from the rapid release of intracellular metabolites from lysed malignant cells and is most frequently associated with hematological malignancies after the initiation of cytotoxic treatment [82]. However, TLS may be delayed, occurring one month or more after the CD19 CAR T cell infusion [44]. TLS has been managed successfully by standard supportive therapy, including allopurinol, hydration, alkalinization, and rasburicase [83]. CRS that is induced by CAR T cell therapy was recently reviewed [84]. CRS is a disorder characterized by nausea, headache, tachycardia, hypotension, rash, and shortness of breath caused by the release of cytokines from the immune cells. CRS is also frequently observed in the clinical trials that treat hematological malignancies with CAR T cells. Severe CRS is described as a cytokine storm, which can be fatal [30]. CRS usually occurs 6–20 days after the infusion of CAR T cells, although it may occur in a very short time in some patients and may be related to various CAR structures, underlying diseases and the patients’ genetic polymorphisms. The current management of CRS includes corticosteroids, cytokine antagonists and supportive therapy [84]. In addition, CRS-related mortality should be reduced by designing safer CARs, following a strict dose-escalation scheme, intensively monitoring the inflammatory cytokines and taking timely and effective measures, including the administration of various antagonists of cytokines under the current situation [84].

In addition to these toxicities, anaphylaxis has been reported in patients infused with CAR-T cells. Maus et al. [85] reported the safety observed in four patients treated with mRNA electroporated murine anti-human mesothelin CAR T cells. One subject developed anaphylaxis and cardiac arrest within minutes of completing the third infusion, most likely because it induced an IgE antibody specific for the murine-based antibody sequences present in the CAR-modified T-cell product. These results indicate that the potential immunogenicity of CARs derived from murine antibodies may be a safety issue for mRNA CARs, especially when administered using an intermittent dosing schedule.

### Blocking inhibitory molecules

Despite encouraging results in clinical trials, the existence of a number of different immunosuppressive pathways can limit the full potential of CAR T cell therapies. The interaction of inhibitory molecules on activated T-cells and their ligands on tumor cells compromises T-cell function. This includes the increased expression of inhibitory immune receptors such as T-cell membrane protein-3 (TIM-3), cytotoxic T lymphocyte-associated antigen 4 (CTLA-4), and/or programmed death-1 (PD-1) on T cells following T-cell activation, which can limit the duration and strength of the adaptive immune response [86].

Promising clinical results infusing mAbs that block the interaction between PD-1 and PD-L1 (or PDL2) in patients with solid tumors have been reported [87, 88]. Thus, blocking this pathway may further enhance the antitumor activity of the gene-modified T cells. Indeed, John et al. [89] first showed that the expression of the PD-1 receptor was significantly increased on Her2 CAR T cells following its coculture with PD-L1+ Her-2+ expressing tumor targets. They further demonstrated that the administration of an anti-PD-1 antibody can significantly enhance the therapeutic efficacy of CAR T-cell therapy in vivo. On September 4, 2014, U.S. Food and Drug Administration (FDA) approved anti-PD-1 antibody pembrolizumab for the treatment of patients with unresectable or metastatic melanoma. These results are encouraging for moving towards testing this combined approach in a clinical setting.

### Conclusions and future directions

In conclusion, our review discussed the development of CAR technology and highlighted some key issues for avoiding the severe adverse events of CAR T cells-based therapy. The judicious selection of candidate TAAs is essential for improving efficacy and safety. Factors that require further consideration include the CAR design, the affinity of the scFv, the density of target molecules, disease burden, the route of administration of CAR T cells and the tumor microenvironment.

CAR-based ACT has emerged as a promising immunotherapeutic strategy and already has shown impressive success, particularly for patients with hematological malignancies. Currently, investigators are extending this strategy to solid tumors [90]. Genetic engineering strategies can meet some of the requirements for an effective CAR-based therapy [2], which includes enabling T cells/NK cells to respond more powerfully against tumor cells and facilitating trafficking to tumors and persistence for long periods. To maximize therapeutic safety, introducing a controllable suicide gene such as an inducible caspase-9 (iCasp9) as a safety switch may increase the safety of cellular therapies and expand their clinical applications. With further modifications in the laboratory and an increased number of clinical trials to test this strategy, engineered CAR-based ACT for cancer may provide significant improvements in cancer immunotherapy.

## Abbreviations

CARs: Chimeric antigen receptors
ACT: Adoptive cellular therapy
TILs: Tumor-infiltrating lymphocytes
TM: Transmembrane
mAb: Monoclonal Antibody
TAAs: Tumor-associated antigens
HAMA: Human anti-mouse antibody
TLS: Tumor lysis syndrome
CRS: Cytokine release syndrome
TIM-3: T-cell membrane protein-3
CTLA-4: Cytotoxic T lymphocyte-associated antigen 4
PD-1: Programmed death-1.
